# Suitable parameter choice on quantitative morphology of A549 cell in epithelial–mesenchymal transition

**DOI:** 10.1042/BSR20150070

**Published:** 2015-06-11

**Authors:** Zhou-Xin Ren, Hai-Bin Yu, Jian-Sheng Li, Jun-Ling Shen, Wen-Sen Du

**Affiliations:** * Institute of Gerontology, Henan University of Traditional Chinese Medicine, Longzihu University Park, Zhengzhou, Henan 450046, China; † First Affiliated Hospital of Henan University of Traditional Chinese Medicine, Zhengzhou, Henan 450000, China; ‡ Affiliated Hospital of Henan Academy of Traditional Chinese Medicine, Zhengzhou, Henan 450004, China

**Keywords:** A549 cell, EMT, evaluation, morphological parameters

## Abstract

Evaluation of morphological changes in cells is an integral part of study on epithelial to mesenchymal transition (EMT), however, only a few papers reported the changes in quantitative parameters and no article compared different parameters for demanding better parameters. In the study, the purpose was to investigate suitable parameters for quantitative evaluation of EMT morphological changes. A549 human lung adenocarcinoma cell line was selected for the study. Some cells were stimulated by transforming growth factor-β1 (TGF-β1) for EMT, and other cells were as control without TGF-β1 stimulation. Subsequently, cells were placed in phase contrast microscope and three arbitrary fields were captured and saved with a personal computer. Using the tools of Photoshop software, some cells in an image were selected, segmented out and exchanged into unique hue, and other part in the image was shifted into another unique hue. The cells were calculated with 29 morphological parameters by Image Pro Plus software. A parameter between cells with or without TGF-β1 stimulation was compared statistically and nine parameters were significantly different between them. Receiver operating characteristic curve (ROC curve) of a parameter was described with SPSS software and *F*-test was used to compare two areas under the curves (AUCs) in Excel. Among them, roundness and radius ratio were the most AUCs and were significant higher than the other parameters. The results provided a new method with quantitative assessment of cell morphology during EMT, and found out two parameters, roundness and radius ratio, as suitable for quantification.

## Abstract (precis)

Evaluation of morphological changes in cells is key for study of epithelial to mesenchymal transition. In the paper, parameters for quantitative evaluation of the changes were explored and two suitable parameters were found, roundness and radius ratio.

## INTRODUCTION

Epithelial to mesenchymal transition (EMT) plays an important role in the metastasis of lung cancer, and has great therapeutic interest in the treatment of cancer [1,2], etc. As a human lung adenocarcinoma cell, A549 cell has been applied widely in EMT study [3,4]. Transforming growth factor-β1 (TGF-β1), one of potent and well-studied cytokine, has been shown to alter the morphology of A549 cell line from cobblestone shape to elongated spindle shape, a fibroblastoid appearance [5–8]. The morphological change has been an important parameter in EMT studies, widely used for assessing anti-EMT effect of compounds, natural products [9,10], etc. However, in most studies, the morphological change was directly qualitative description [5–8,11], seldom evaluation by quantitative parameter. Only in few papers, some quantitative parameters were used for the morphometry, including cellular size, roundness [12,13], but we did not find a report about suitable parameter choice on quantitative morphology of A549 cell in EMT.

For accurately evaluating A549 cellular changes in EMT, exact acquisition of cellular shape and suitable morphological parameters are essential. At present, some image analysis softwares are convenient for not only in acquisition of exact cell shape but also in quantitative analysis of cellular morphology by many parameters. In Adobe Photoshop software, some techniques, including of colour contrast, image adjust, histogram commands, magic wand for select similar, grayscale absorbance and images merging, have been used in quantification of different chromogens in double immunohistochemical staining, of hormone receptors in breast cancer and a new image creation by merging several images for covering entire tumour area [14–16]. And in Image Pro Plus software, techniques of HSI (hue–saturation–intensity) representation, area as colour score, colour enhancement and threshold, background flattening, have been applied in quantification of molecular stress in cetaceans and of tissue infiltrating leucocytes [16,17]. Furthermore, Image Pro Plus software has dozens parameters for morphometry analysis, reflecting character of different shapes.

In the study, we planned to acquire exact shape of A549 cells both stimulated by TGF-β1 and un-stimulated by TGF-β1 with Photoshop software, then, calculate parameters of the cells with Image Pro Plus software, finally, compare difference of the parameters. We expected to find out some suitable parameters for the quantitative morphology.

## MATERIALS AND METHODS

### Cell culture and image acquisition

A549 cell, the human alveolar epithelial carcinoma cell line, was purchased from the Institute of Biochemistry and Cell Biology of Shanghai Institutes for Biological Sciences of Chinese Academy of Sciences. According to previous reports [18,19] and our practice, EMT of cells was induced by TGF-β1. Cells were cultured in RPMI1640 medium (Beijing Solarbio Science & Technology Co., Ltd) supplemented with 10% fetal bovine serum (Tian Hang Biological Technology Co., Ltd) at 37°C in a humidified 5% CO_2_ atmosphere. Confluent cultures of cells were maintained in serum-free RPIM for 12 h before treatment with recombinant human TGF-β1 (PeproTech, Catalog#100-21). Then medium was removed. Some cells were added RPMI1640 medium supplemented with 10% fetal bovine serum and 5.0 ng/mL TGF-β1 and other cells were added RPMI1640 medium supplemented with 10% fetal bovine serum. Subsequently, all cells were cultured for 48 h. After culture, cells were placed in phase contrast microscope (Olympus CKX41-A32PH) and three arbitrary fields of a well in a plate were captured by digital camera (Olympus C5060 Wide Room), and saved in a personal computer.

### Acquisition of cells shape with Adobe Photoshop software

**Figure 1:**
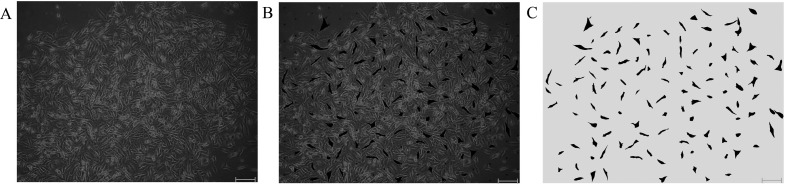
Choosing, shape depiction and segmentation of cells in images (**A**) An original image stimulated by TGF-β1. (**B**) The image with points of grid and chosen cells filled with black. (**C**) Segmented cells in the image stimulated by TGF-β1. The images were captured by phase contrast microscope (Olympus CKX41-A32PH, Tokyo, Japan) with a CPLN10XPH 10×/0.25 NA objective (bars, 40 μm).

Adobe Photoshop CS2 (Adobe Systems Inc.) software was used for image process. As a first step, the images were set to be same size and a blank image with same size as above images was established. In the blank image, grids were appeared, then, cross points of the grids were coloured in black with Brush tool. Subsequently, applying Image tool was used to merge the image into a cellular image to add cross points into the image. In the step, point density is essential for selecting cell amounts. In Gridline every box of Grid and Slices menu, the chosen number decided the point density. Large number produced high point density and small number produced low point density. The set of size and colour selection of the points was another key point. Proper colour should be very distinct to be identified out of an image and proper size should be easy to find and should never disturb cellular depiction. Cells with at least one cross point were selected for next cellular shape acquisition, and Paths of Pen tools was used to depict cell shape, enclose border line of the cell outline, fill unique hue into all selected cells. After all cells areas were filled with one colour, they were distinct with other cells and other part in the image, were easy to be chosen with Magic Wand tool, subsequently, the other part of the image was chosen by Inverse tool and the area of the part were changed into another unique hue (Figure 1). Finally, the images were saved as JPG image format for following morphological analysis.

**Table 1:** Definition of parameters in image pro plus software

Parameters	Definition
Angle	Between the major axis of the object and the vertical
Area	Area of object. Does not include holes’ area if <Fill Holes> option is turned off
Area (polygon)	Area included in the polygon defining the object’ outline. Some polygon as that used for perimeter
Area/box	Ratio between area of object and area of its bounding box
Aspect	Ratio between major axis and minor axis of ellipse equivalent to object
Axis (major)	Length of major axis of ellipse with same moments of order 1 and 2 as object
Axis (minor)	Length of minor axis of ellipse with same moments of order 1 and 2 as object
Box height	Height of the object's bounding box
Box width	Width of the object's bounding box
Box *X*/*Y*	Ratio between width and height of object's bounding box
Centre-*X*	*X* coordinate of object's centroid
Centre-*Y*	*Y* coordinate of object's centroid
Diameter (max)	Length of longest line joining two points of object's outline and passing through the centroid
Diameter (mean)	Average length of diameters measured at 2 degree intervals and passing through object's centroid
Diameter (min)	Length of shortest line joining two points of object's outline and passing through the centroid
Feret (max)	Longest caliper (feret) length
Feret (mean)	Average caliper (feret) length
Feret (min)	Smallest caliper (feret) length
Fractal dimension	Fractal dimension of the object's outline
Perimeter (convex)	Perimeter of the convex outline of the object
Perimeter (ellipse)	Perimeter of the equivalent ellipse
Perimeter (ratio)	Ratio of convex perimeter to perimeter
Radius (max)	Maximum distance between object's centroid and outline
Radius (min)	Minimum distance between object's centroid and outline
Radius ratio	Ratio between max radius and min radius
Roundness	Perimeter^2^/(4 × *π* × area)
Size (length)	Feret diameter along major axis of object
Size (width)	Feret diameter along minor axis of object

### Calculation of morphological parameters of cells in processed images

Thirty TGFβ1-treated images and thirty TGFβ1-untreated images were given for measure. After the images were processed by Photoshop according to above methods, cells in the final processed images were calculated morphological parameters by Image Pro Plus 6.0 software (Media Cybernetics) as following.

Open images in Image Pro Plus software, then click Measure in menu bar; click Count/Size in Measure menu and open the Count/Size dialogue box; choose Manual and click Select Colors, then enter into Segmentation dialogue box. In the box, Eyedropper colour selection tool is clicked, then, the cell area is clicked at random for selecting all cells area. Subsequently, click Close button and return Count/Size window. In the window, Measure menu is clicked and Select Measurements is chosen; in the dialogue box, click parameters then click OK and return Count/Size dialogue box, at last, click Count and finish calculation of cells. The results can be found in Measurement Data in View of Count/Size.

In Measure menu, there are rich parameters for morphometry calculation and analysis, reflecting various characters of different shapes. Information regarding the parameters used in the study is summarized in Table 1.

**Figure 2:**
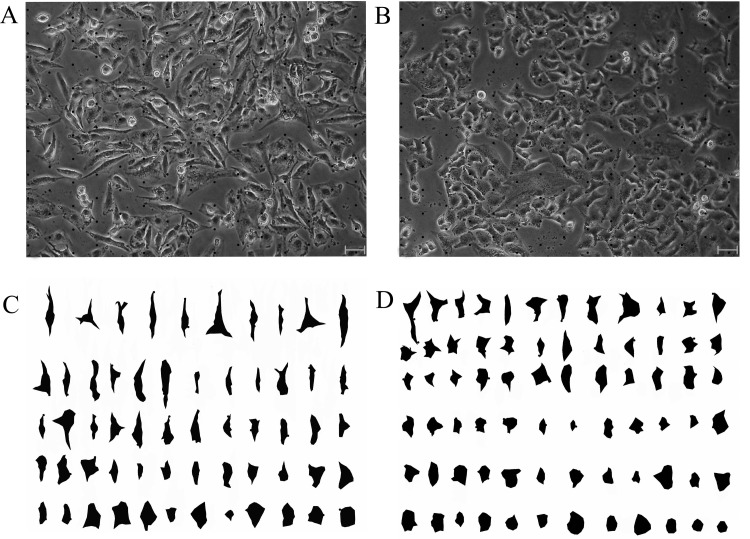
Images of cell shape in segment pre-and post (**A**) An image with points of grid stimulated by TGF-β1. (**B**) An image with points of grid never stimulated by TGF-β1. (**C**) The image stimulated by TGF-β1 with segmented and sorted cells. (**D**) The image stimulated never by TGF-β1, with segmented and sorted cells. The images were captured by phase contrast microscope (Olympus CKX41-A32PH, Tokyo, Japan) with a LCACHN20XPH 20×/0.4 NA objective (bars, 20 μm).

Furthermore, cells in an image were arrayed as roundness by using Sort Objects technique in the software, which gives us advantageous for observing cellular shape character (Figure 2).

### Statistical analysis

SPSS16.0 was used for statistical analysis. One-Sample Kolmogorov–Smirnov Z in nonparametric tests was applied for data distribution, and found these data were no normal distribution (results never showing). Two-Sample Kolmogorov–Smirnov test in nonparametric tests was used to evaluate the difference of a parameter between TGF-β1-untreated images group and TGF-β1-treated images group. Areas under the curves (AUCs) of receiver operating characteristic curve (ROC curve) of a parameter was described with SPSS software and *F*-test was used to compare two parameters AUCs in Excel. *P*< 0.05 was considered significant.

## RESULTS

### Results of image processing of Adobe Photoshop software

Most untreated A549 cells showed a pebble-like shape and cell–cell close adhesion and most TGF-β1-treated cells showed a decrease in cell–cell contacts and adopted a more elongated morphological shape. An image had many cells. Therefore, some of them should be selected for analysis. With assist of cross points, an observer is convenient to find out the analysed cells without subjectivity. Subsequently, outline depiction, area filled with unique hue of the cells and hue shift in the background of the image were carried out. The new image had only two colours, one for cells and another for background, increasing efficiency, accuracy for following cellular morphological analysis (Figure 1).

### Comparison of values of morphological parameters of cells between TGF-β1-treated and TGF-β1-untreated A549 cells

**Figure 3:**
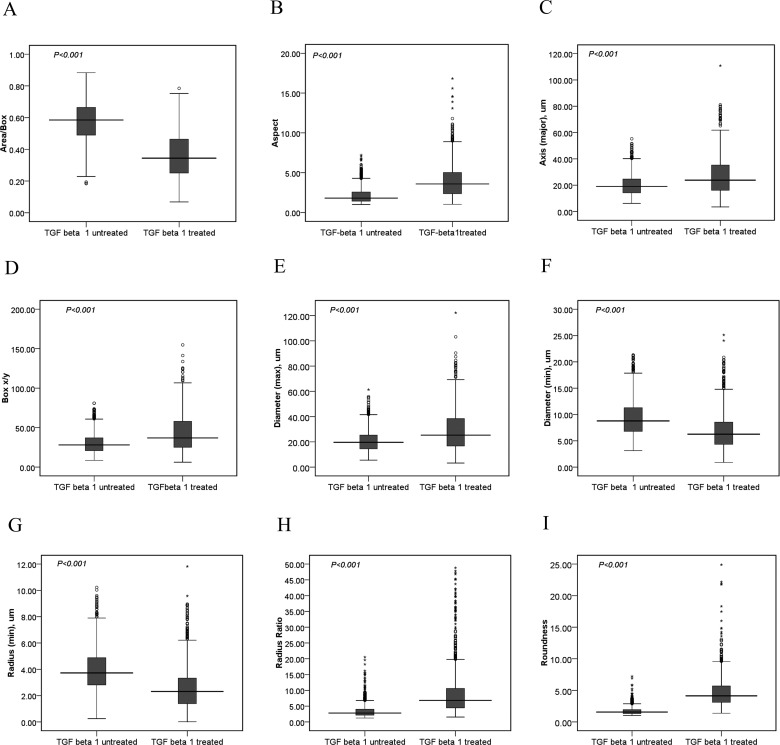
Nine parameters on cellular shape compared between TGF-β1-treated group and TGF-β1-untreated group Between TGF-β1-treated group (cell numbers=1267) and TGF-β1-untreated group (cell numbers=1832), nine morphological parameters as area/box (**A**), aspect (**B**), axis major (**C**), box *X*/*Y* (**D**), diameter max (**E**), diameter min (**F**), radius min (**G**), radius ratio (**H**) and roundness (**I**) showed statistically difference (*P*< 0.001) with two-sample Kolmogorov–Smirnov Test in nonparametric tests by SPSS 16.0 software.

One thousand two hundred sixty-seven cellular shapes from 30 TGF-β1-treated images and 1832 cellular shapes from 30 TGF-β1-untreated images were calculated with different morphological parameter values by Image Pro Plus 6.0 software. Between the TGF-β1-treated group and TGF-β1-untreated group, difference in nine parameters was significant (*P*< 0.001, Figure 3).

### AUCs of parameters and comparison of the parameters for reflection on EMT changes

In a word, more high a AUCs of a parameter is, more accurate the parameter is [1,5]. The sequence of AUCs in the nine parameters, from high to low, was roundness, radius ratio, aspect, area/box, axis major, diameter max, radius min, diameter min, box *X*/*Y*. Here, the value of box *X*/*Y* was approximately 0.5, showing the parameter never suitable for reflection on EMT changes. *F*-test was used for assessing the statistical difference of AUCs between any two parameters for finding the best parameter for EMT changes. Among them, roundness and radius ratio were the maximum value of AUCs and the values were significantly higher than other parameters (*P*< 0.002–0.000) (Tables 2 and 3).

**Table 2:** Values of nine parameters on AUCs and S.E.M.

Parameters	AUCs	S.E.M.
Aspect	0.8145	0.0079
Box *X*/*Y*	0.5039	0.0113
Axis (major)	0.7073	0.0097
Diameter (max)	0.7050	0.0098
Radius ratio	0.8446	0.0070
Roundness	0.8600	0.0067
Area/box	0.8076	0.0080
Diameter (min)	0.6428	0.0102
Radius (min)	0.6820	0.0099

**Table 3:** Comparison of AUCs *P*-value in *F*-test between any two parameters of nine parameters

Parameters	Radius ratio	Aspect	Area box	Axis (major)	Diameter (max)	Radius (min)	Diameter (min)	Box *X*/*Y*
Roundness	0.112	0.000	0.000	0.000	0.000	0.000	0.000	0.000
Radius ratio	–	0.002	0.000	0.000	0.000	0.000	0.000	0.000
Aspect	–		0.270	0.000	0.000	0.000	0.000	0.000
Area/box	–			0.000	0.000	0.000	0.000	0.000
Axis (major)	–				0.436	0.034	0.000	0.000
Diameter (max)	–					0.049	0.000	0.000
Radius (min)	–						0.003	0.000
Diameter (min)	–							0.000

## DISCUSSION

Many studies reported stimulated by TGF-β1, A549 cellular morphological change was significant [5–8]. For assessment for EMT deterioration, cellular morphometry is low cost, simple operation, and easy training for new observers. Therefore, it has been a general index applied in effect assessment of anti-fibrosis and anti-cancer drugs [5–8]. However, in most studies, the morphological changes of cells were directly described in words, never valuated by quantitative value. Only few papers, some quantitative parameters were used for cell morphometry, including cellular size, roundness [12,13]. However, for A549 cellular morphological analysis, few researches explored to valuate in which parameters were suitable for qualifying the EMT changes.

To quantitatively explore cellular morphological change in EMT, it is necessary to objective acquisition of a plenty of cell amount, accurate abstraction of cellular shape. But complex image background often makes observers vision fatigue, disturbing observers to accurately describe or acquire cells shape. So in some studies, some methods were employed to remove or decrease background noise and Photoshop software had been employed for the purpose [20,21]. Another problem is what to solve subjectivity of choosing cells for analysis from complex images. Some method was given to solve the problem. For example, double square lattice-B100 was placed on the screen of the projection microscope and manually evaluated the morphometric parameters, which increase objective for chosen cells from an image and decrease time on choosing cells [12]. In the study, images merging technique in Photoshop software was employed for adding assistant-points in images, giving objective, convenient way for choosing cells. Furthermore, the chosen cells were depicted their outline, filled area with unique hue and other part in the image was filled into another hue. The new images were only two colours, cells with one hue and background with another hue, and were advantageous for Image Pro Plus software to segment automatically cells area for parameter calculation due to no overlapping between cell hue and background hue. Observing the cells shape, cells both normal or EMT images had various shapes, for example, cobblestone shape and elongated spindle shape were not only in normal images but also in EMT images. Therefore, qualitative morphological analysis, especially with words depiction, was not exact and quantitative morphology was necessary for reflecting the shape change.

Image Pro Plus software, a commercial general purpose image processing software, has dozens of parameters to detect cellular shape. In the study, 29 parameters were employed to compare cellular morphology between in normal images and in EMT images. Among them, difference of nine parameter values was significant.

As a statistical tool, the ROC curve is employed for evaluating the accuracy of diagnostic tests and binary classifiers [22,23]. Accuracies of several biomedical parameters could be compared with their AUCs, an important and widely used index [23]. The higher AUC value of a parameter shows that the parameter is higher accurate for a diagnostic test, suggesting the parameter is more probable as an appropriate index. The above nine parameters were calculated in AUCs of ROC curves. The AUC of box *X*/*Y* was only 0.5039, showing no value for the cellular morphology change. The AUCs of axis major, diameter max, radius min, diameter min were from 0.6428 to 0.7073, showing not suitable parameters. Roundness, radius ratio, aspect, area/box were approximately 0.8, showing possibility as suitable parameters. Among them, roundness and radius ratio were the most value of AUCs and the values were significantly higher than other parameters. Moreover, the values between roundness and radius were never significant. The results showed that the two parameters were the most accuracy reflection of EMT changes. Roundness reflects the level of cellular area resembling circle area, and radius ratio is ratio between max radius and min radius of a cell shape. In normal cells, many cells were cobblestone similar to circle shape, and in EMT cells, many cells were elongated spindle shape with high ratio of max radius to min radius. So the two parameters are good parameters for reflection of morphological changes from normal cells to EMT cells.

Usually, various shapes are in a large number of cells in a micro-image. So it is essential for collection of different parameters for reflecting morphological character of cells in the image. For example, shape, size of cell area and branch numbers of nuclear and so on, are extracted and collected for leucocytes classification. In EMT, cell morphological change mainly should include epithelial-shaped cells percent decrease and mesenchymal-shaped cells percent increase. Single roundness parameter used for reflecting morphological character of cells is partial, low accuracy. Adding radius ratio parameter could reflect morphological change of mesenchymal cells. We hypothesize that threshold or reference range of different parameters including of roundness, radius ratio and other parameters should be helpful for evaluating EMT appearance, severity.

In the study, we established a set of methods of quantitatively cellular morphology in EMT cells. Cells selecting and parameters calculation were fast speed, self-automation. However, cellular outline depiction was low speed, manual, remained a bottle-neck for the whole methods. In a next step, we will improve the depiction and expect to find a better way.

## Abbreviations

AUC: areas under the curve
EMT: epithelial to mesenchymal transition
ROC: receiver operating characteristic
TGF-β1: transforming growth factor-β1
